# Major Challenges and Potential Microenvironment-Targeted Therapies in Glioblastoma

**DOI:** 10.3390/ijms18122732

**Published:** 2017-12-16

**Authors:** Ali S. Arbab, Mohammad H. Rashid, Kartik Angara, Thaiz F. Borin, Ping-Chang Lin, Meenu Jain, Bhagelu R. Achyut

**Affiliations:** Tumor Angiogenesis laboratory, Georgia Cancer Center, Department of Biochemistry and Molecular Biology, Augusta University, Augusta, GA 30912, USA; aarbab@augusta.edu (A.S.A.); mrashid@augusta.edu (M.H.R.); kangara@augusta.edu (K.A.); tborin@augusta.edu (T.F.B.); plin@augusta.edu (P.-C.L.); jainm25@hotmail.com (M.J.)

**Keywords:** tumor microenvironment, Glioblastoma, neovascularization, antiangiogenic therapy, resistance, bone marrow-derived cells, myeloid cells

## Abstract

Glioblastoma (GBM) is considered one of the most malignant, genetically heterogeneous, and therapy-resistant solid tumor. Therapeutic options are limited in GBM and involve surgical resection followed by chemotherapy and/or radiotherapy. Adjuvant therapies, including antiangiogenic treatments (AATs) targeting the VEGF–VEGFR pathway, have witnessed enhanced infiltration of bone marrow-derived myeloid cells, causing therapy resistance and tumor relapse in clinics and in preclinical models of GBM. This review article is focused on gathering previous clinical and preclinical reports featuring major challenges and lessons in GBM. Potential combination therapies targeting the tumor microenvironment (TME) to overcome the myeloid cell-mediated resistance problem in GBM are discussed. Future directions are focused on the use of TME-directed therapies in combination with standard therapy in clinical trials, and the exploration of novel therapies and GBM models for preclinical studies. We believe this review will guide the future of GBM research and therapy.

## 1. Glioblastoma Statistics

Glioblastoma (GBM) is the most common highly malignant adult primary intracranial neoplasm. GBMs comprise 14.9% of all primary brain and central nervous system (CNS) tumors, 47.1% of the malignant primary brain and CNS tumors, and 56.1% of all gliomas. According to the 2017 Central Brain Tumor Registry of the United States (CBTRUS) report, the average annual age-adjusted incidence rate of GBM is 3.20/100,000 population. The incidence rate ratio is significantly high in older ages [1], in males compared to females (1.58) and in whites compared to blacks (1.93). In past years, GBM had the highest number of cases of all malignant CNS tumors, with 12,500 cases projected in 2017 and 12,760 in 2018 [2]. Overall, GBM has a poor prognosis with quite low relative survival estimates; only 5.5% patients between the age of 55–64 survive five years [2]. 

## 2. Overarching Challenges

GBM tumors harbor a large network of blood vessels, have invasive features, and exhibit severe hypoxia with highly complex genetic, molecular and cellular mechanisms. All of these hallmarks contribute to therapy resistance and tumor recurrences, a common outcome was seen in the clinic. In the following sections, we will discuss current challenges in GBM therapy including tumor cells extrinsic characteristics such as the myeloid cell-rich tumor microenvironment (TME) and tumor cell intrinsic properties such as the genetic and molecular heterogeneity that drives therapy resistance. The potential combination of microenvironment-targeted therapies with standard therapies, which could be a key in future GBM therapy, will be discussed at the end. Moreover, this article will highlight critical and translational aspects of GBM.

### 2.1. Challenges Due to Hypoxia and Hyper-Vasculaturity in the Microenvironment

GBMs are considered to be hypoxic solid tumors. When the tumor grows larger than a critical size (2–3 mm in diameter), it cannot cope with the nutritional demand of the rapidly dividing and growing cancer cells, leading to hypoxia. Hypoxia is one of the major challenges in GBM therapy [3]. In addition, GBM is the most vascularized CNS cancer with the highest degree of vascular proliferation and endothelial cell hyperplasia [4]. One of the classical mechanisms, angiogenesis, which is the formation of new blood vessels, plays a pivotal role in GBMs development and growth. GBM vasculatures are functionally and structurally anomalous; they are characterized by coarse vessel diameter, permeability, tortuosity, and thickened basal lamina that can also lead to more hypoxic regions [5]. Several proteins, cytokines, and factors are known to contribute to the GBM microenvironment (Table 1) [6,7]. Studies have investigated that tumor-associated hypoxia results in upregulation of hypoxia-inducible factor-1α (HIF-1α), which subsequently leads to upregulation of several molecular mediators, e.g., vascular endothelial growth factor (VEGF). VEGF family members signal predominantly through the receptor tyrosine kinases, VEGF receptors (VEGFR)-1, VEGFR-2, and VEGFR-3, in association with the co-receptors [8]. HIF-1α contributes to induction of stromal cell-derived factor 1 (SDF1α) in the TME, which helps in recruiting vascular modulatory bone marrow-derived cells (BMDCs) to stimulate angiogenesis, vasculogenesis, invasion, and immunosuppression mechanisms in GBM tumors [9]. 

In addition to VEGF and SDF1α, other pro-angiogenic factors upregulated in GBMs include hepatocyte growth factor (HGF), fibroblast growth factor (FGF), platelet-derived growth factor (PDGF), placenta-like growth factor (PLGF), angiopoietin-2 (ANGPT2), interleukin-8 (IL-8), matrix-metalloproteinase (MMP)-2, MMP-9, collagen type I α1 (COLIA1), endothelial markers CD34, Tenascin-C, neuron-glial antigen 2 (NG-2) on pericytes, insulin-like growth factor (IGF), and epidermal growth factor (EGF). VEGF is one of the most crucial growth factors and plays an indispensable role in GBM neovascularization by interacting with a number of signaling pathways to advance GBM growth [10]. These pathways include activation of RAS/RAF/mitogen-activated protein kinase (MAPK) [11,12], phosphatase and tensin homolog (PTEN)/phosphoinositide (PI) 3-kinase/AKT [13], phospholipase C-γ/protein kinase C [13], nitric oxide (NO) [14], platelet-derived growth factor (PDGF)-B [15], and the notch–delta-like ligand (DLL) 4 signaling pathway [16]. GBMs are diagnosed at the advanced stages when they harbor hypoxia and leaky vasculatures. Therefore, we need adjuvant treatments that have capabilities (1) to normalize blood vessels (2) that can penetrate hypoxic regions of the GBM tumor along with current standard therapies and (3) that can block the infiltration of BMDCs and myeloid cells to the GBM tumors, overcoming the challenges in GBM therapies. Interestingly, heterogeneity exists in context to hypoxia and angiogenic areas within the tumors. At the invading front, tumors exhibit hypoxic stress and promote neovascularization. A recent study discovered that tumors display distinct metabolic profile depending on the microenvironment [17]. The dynamic plasticity in metabolism could have implication in therapeutic failures seen in anti-GBM therapies in the past.

### 2.2. Challenges Due to Microenvironment-Driven Resistance to Antiangiogenic Therapy

For the first time, Folkman et al. demonstrated that tumor growth was dependent on continual blood supply and rapid vascularization, a process called angiogenesis. It was believed that targeting pathological angiogenesis would curb the growth of the tumor by impeding the formation of tumor-associated neovessels [18,19]. The idea of angiogenesis driving tumor growth and metastasis gained momentum and soon the concept of tumor angiogenesis became a hallmark of solid tumors [18,20,21,22]. The hypervascular nature of GBM tumors prompted the implementation of anti-angiogenic therapy (AAT) as an adjuvant to the current standard of care, which included surgical resection of tumors, radiotherapy (RT), and temozolomide (TMZ) chemotherapy. Drugs targeting the VEGF–VEGFR pathway, such as Vatalanib, Sunitinib, and Cediranib found therapeutic application in the treatment of many hypervascular solid tumors including GBM [23,24,25,26,27,28]. GBM tumors are extremely heterogeneous and targeting the endothelial cells was believed to be a viable option for countering the uncontrolled tumor growth. However, GBM tumors developed refractoriness and therapeutic resistance to AAT, which was observed in many GBM cases, thereby limiting the temporal benefits of AAT [29]. Previous magnetic resonance imaging (MRI) studies from our laboratory reported that employing the VEGFR2 inhibitor Vatalanib (PTK787) caused a significantly larger GBM tumor [30]. Vatalanib treatment induced hypoxia and was associated with the increased expression of several pro-angiogenic cytokines and chemokines such as VEGF, SDF-1, HIF-1α, FGF-1, FGF-2, Ephrin (Eph)-A1, Eph-A2, Angiopoietin-1, and their corresponding receptors VEGFR2, VEGFR3 and EGFR at the tumor-invading front of GBM [31]. Vatalanib, Cediranib and Sunitinib, and inhibitors of VEGFR, PDGFR, and c-kit receptors have offered limited therapeutic efficacy and transient benefits and have been associated with higher toxicities in clinical trials [32,33,34,35,36]. The research groups that propagated the idea of AAT did not account for the involvement of BMDCs in the formation of tumor neovasculature through vasculogenesis in GBM.

A recent study from our laboratory demonstrated that Vatalanib treatment increased the number of CD68+ myeloid cells as well as the CD133+, CD34+, and Tie2+ endothelial cell signatures in a novel chimeric mouse model of GBM. As a part of that study, we sought to explore the effects of AAT on the recruitment of BMDCs. We found that GFP+ chimeric bone marrow cells co-localized at the tumor periphery with VEGF, SDF-1α, and PDGF. In addition, our study found a significantly higher number of GFP+ bone marrow cells in close proximity to CD11b+ and F4/80+ cells [37]. In another study, we reported that the paradoxical growth of tumor following Vatalanib treatment was controlled by inhibiting the mobilization of BMDCs and disrupting the CXCR4-SDF-1α interaction using whole body irradiation and AMD3100, respectively [38]. BMDCs orchestrated the therapeutic resistance to AAT in a GBM model. The TME was identified as highly immunosuppressive and pro-angiogenic in GBM. Achyut et al. [39] demonstrated the therapeutic utility of CSF1R inhibitor in controlling AAT resistance in GBM models. The authors established an extracellular signal-regulated kinase (ERK)-mitogen-activated protein kinase (MAPK)-mediated upregulation of CXCL7 following AAT in a GBM model. By employing a CSF1R inhibitor (GW2580), the authors reported a decrease in the levels of CXCL7 thereby establishing a novel cytokine-dependent pathway for controlling AAT resistance in GBM. Further, the authors found that the NFκB pathway was central to the chemokine axis driving GBM growth and conditional deletion of the p65 subunit of the NFκB transcription factor inhibited GBM tumor growth in an immune competent model, suggesting a critical role of inflammation in GBM pathogenesis [40].

Bevacizumab, a humanized monoclonal antibody against VEGF-165, the predominant isoform of VEGF-A, was the first angiogenesis inhibitor to obtain Food and Drug Administration (FDA) approval in 2004 and was the first commercially successful antiangiogenic drug marketed [41]. Although the use of bevacizumab in combination with chemotherapy resulted in some dramatic tumor size reduction with prolonged progression-free survival [42], prolonged use led to deteriorated clinical outcome and development of therapeutic resistance [43,44]. Despite somewhat prolonged progression-free survival, treatment with lomustine plus bevacizumab failed to confer a survival advantage over treatment with lomustine alone in patients with progressive GBM in a European clinical trial [45]. The development of therapeutic resistance in GBM is primarily attributed to the activation of alternative pathways of neovascularization to counter the therapeutic insult [46,47]. Recently, Wang et al. summarized all the AAT clinical trial data and proposed the targeting of alternate pro-angiogenic mechanisms in GBM [5]. Moreover, all the clinical and preclinical data indicate that use of any class of AAT is not an ingenious choice for GBM therapy.

### 2.3. Challenges Due to Microenvironment-Driven Alternative Vascularization 

The solid tumors have the capability to sustain and grow through complex networks of neovascularization. The tumor vasculature was believed to encompass angiogenesis process of endothelial sprouting and proliferation as a principal route of new blood vessel formation. Recent data suggest that tumors have several distinct mechanisms of neovascularization to drive tumor growth, such as vasculogenesis and vascular mimicry (VM) [48]. VM phenomenon was identified as one of the key tumor-inherent mechanisms to drive AAT resistance in GBM tumors [49,50,51]. VM is the uncanny ability of tumor cells to transdifferentiate into endothelial-like phenotypes and form neovascular structures to irrigate the hypoxic tumors to meet the nutritional and metabolic demands [52,53]. Tumor cells switch to adaptive mechanisms of neovascularization under a prolonged hypoxic environment at the tumor center to counter the selective pressure exerted upon the tumor cells induced by AAT. These mechanisms include upregulation of pro-angiogenic growth factors, chemokines, and cytokines necessary for sustaining the growth of tumor cells as well as for the recruitment of endothelial progenitor cells (EPCs), endothelial cells (ECs), angiogenic myeloid cells, and vasculogenic leukocyte cells [50]. Apart from the creation of a niche favorable for the growth of the tumor, a myriad of invasive and metastatic gene signatures are also initiated in the tumor and the tumor-associated stromal cells to ensure sustainable conditions for tumor survival during AAT [54,55]. The growing tumor resorts to the development of novel tumor-dependent pathological neovascularization mechanisms for vascular perfusion independent of the endothelial and vasculogenic systems as the dogma states [47,56].

Emerging literature supports that GBM stem-like cells (GSCs) are instrumental in GBM microenvironment [57,58,59,60]. During VM, transdifferentiated GSCs acquire endothelial characteristics and form a pool of cells that initiate and sustain VM. The trans-differentiation of tumor cells into endothelial-like cells, which were CD45− CD31+ CD34+ (termed tumor-derived endothelial cells, TDECs) [61] and GSCs that were CD133+CD144+ [62] initiated and promoted VM in GBM models. GSCs also transdifferentiate into pericytes and targeting GSCs enhanced chemotherapeutic efficacy through disrupting the blood-tumor barrier, indicating a critical role of GSCs in establishing alternative vasculature in growing GBM [57]. VM in gliomas positively correlates with high grades of tumor malignancy and invasiveness is associated with extremely poor prognosis [63,64]. In light of these numerous reports and evidence, delineating the mechanisms of VM to better understand this new facet of AAT resistance in GBM is the need of the hour. Therefore, it becomes imperative for us to look into not just the tumor-associated stromal contribution but also tumor cell-dependent heterogeneous mechanisms that confer GBM a paradoxical growth and sustenance advantage along with the refractoriness and relapse associated with the development of AAT resistance.

### 2.4. Challenges Due to an Immune Suppressive Microenvironment Following Standard Therapy

GBM is an invasive neoplasm with a median survival of three months if untreated [2,65] and approximately only 15 months with standard therapies [66]. GBM’s ability to severely invade and infiltrate normal surrounding tissue oftentimes in integral areas of the brain, including areas that control speech and motor functions make comprehensive resection impossible. Infiltrating tumor cells invariably remain within the surrounding brain, and prompt to later disease progression or recurrence. Tumors smaller than 5–6 cm and those that do not cross the mid-line, and supratentorial (cerebrum) and cerebellar tumors (persuadable to surgical resection) have been associated with favorable outcomes [67,68]. Current standard therapy for GBMs encompasses maximally secured surgical resection followed by concomitant RT and TMZ chemotherapy [69,70].

Until 2005, postoperative RT alone was the prevailing treatment. Stupp et al. in 2005 [66] showed that RT plus concomitant TMZ chemotherapy, an oral second-generation imidazotetrazine derivative or a DNA alkylating agent that exerted its cytotoxic effects by methylation of specific DNA sites, was more effective than RT alone for improving overall survival of patients with GBM tumors. TMZ is the most successful drug that has added several months to the life expectancy of GBM patients. In some cases, despite aggressive treatment, tumors inevitably recur due to the infiltrative nature of GBM, resulting in poor overall survival [71,72,73,74]. Therapy outcomes are even poorer in elderly patients [75,76]. Reviews of molecular-targeted therapies for primary and recurrent GBM have indicated modest benefits in overall survival of 5 to 8 months [77]. 

Chronic inflammation and the release of cytokines following radiation and TMZ therapies cause a recurrence of GBM [78,79,80]. Recurrent GBMs have an increased infiltration of BMDCs in the tumor bulk and infiltrative regions after therapy [81]. The enhanced myeloid cell infiltration in the TME following chemotherapy was associated with the activation of the CSF1–CSF1R pathway [82,83]. After the TMZ chemotherapy, some tumors displayed aggressive immune suppressive features in the tumor [84,85]. Moreover, tumor cell-secreted factors provide a gradient that helps myeloid cell infiltration into the tumor. The infiltrated myeloid cells, in turn, are capable of impairing chemotherapeutic responses [86,87]. In the autonomous mechanism, several tumor p53 (TP53) mutations in the tumor cell compartment also contributed to tumor progression and resistance to TMZ [88,89]. In some cases, the survival advantage conferred by TMZ is associated with methylation of the promoter region of the gene encoding O^6^-methylguanine DNA-methyl transferase (MGMT) [90]. Presence of TP53 mutations in the tumor cell compartment may decrease the TMZ sensitivity by increasing MGMT expression [88]. Thus, one of the current challenges is to enhance the effectiveness of the available standard therapies against GBM.

### 2.5. Challenges Due to Molecular and Genetic Heterogeneity in GBM Tumors

GBM originates from astrocytes or astroglia that reside in the brain and spinal cord. Astrocytes make up the supportive tissue of the CNS. Initially, GBMs were thought to be derived solely from glial cells; however, several pieces of evidence suggest that GBMs usually contain a mix of cell types at multiple stages of differentiation with significant phenotypic variations [91]. GBM is primarily detected in the cerebral hemisphere of the brain but can be found anywhere in the brain or spinal cord. Recent genetic and molecular advances have contributed to a better understanding of GBM pathophysiology and disease subgroups. Although all GBMs are categorized as World Health Organization (WHO) grade IV astrocytoma, they display strong genetic discrepancy and tumor subtypes with genetic alterations.

Gene expression profiling of GBMs identified distinct transcriptional subgroups such as proneural, mesenchymal, neural, and classical [92,93]. In addition to the transcriptional subgrouping of GBMs, GBMs were grouped based on inactivation pattern of tumor suppressor genes and activation of oncogenes. In the 2016 WHO classification of CNS tumors, GBMs were divided into three main groups based on their shared genetic driver mutations in the gene encoding isocitrate dehydrogenase enzyme 1/2 (IDH1/2), which drives distinct growth pattern and behaviors. This granted a persuasive classification based on both phenotype and genotype: (1) IDH-wild-type GBMs (about 90% of cases), (2) IDH-mutant GBMs (about 10% of cases), and (3) not otherwise specified (NOS) GBMs, for those tumors for which full IDH evaluation cannot be performed [94,95,96,97]. A new variant of GBM (IDH-wild-type), called epithelioid GBM, has been added to the classification and has been found mostly in children and young adults [95,98,99].

In addition, several other molecular genetic alterations have been found in GBMs. Primary GBMs show overexpression of epidermal growth factor receptor (EGFR), mouse double-minute 2 (MDM2), mutations of phosphatase and tensin homolog gene (PTEN). High frequency of telomerase reverse transcriptase (hTERT) promoter and absence of IDH1 mutation have also been seen in primary GBMs. The hallmark of secondary GBMs is a mutation in the IDH1, TP53, and α thalassemia/mental retardation syndrome X-linked (ATRX) genes. Recently, the WHO added a rare and controversial subtype of GBM termed “with oligodendroglioma component” (GBM-O) that occurs in younger patients. This subtype often contains the TP53 and IDH1 mutation, lack of EGFR amplification, and lower frequency of PTEN deletions. In GBM-O group, observed genetic heterogeneity displayed longer survival compared to patients with another group of GBMs [1,100,101,102]. In GBM-O tumors, the combined loss of heterozygosity (LOH) on chromosomes 1 p and 19 q were correlated with classic oligodendroglioma morphology and was associated with IDH mutations, TP53 expression, and MGMT promoter methylation status [103,104,105]. Moreover, these reports indicate that GBM is highly heterogeneous in the context of genetic and molecular alterations. This heterogeneous nature of GBM may have a critical role in regulating therapeutic outcomes.

## 3. Potential Adjuvant Therapies against GBM

**Figure 1:**
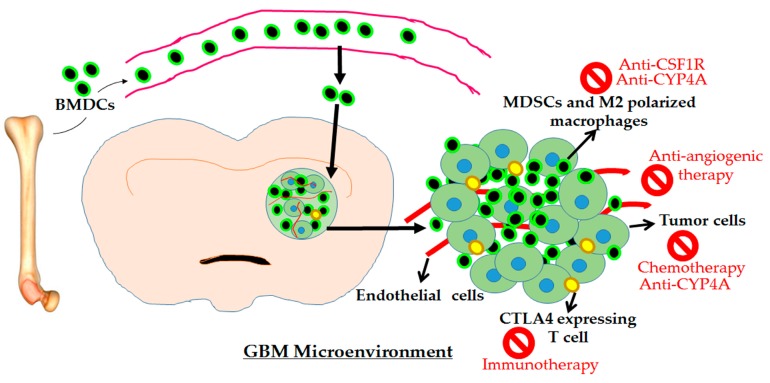
Schematic representation of GBM tumor microenvironment. Bone marrow-derived cells (BMDCs) are recruited to the tumor during the GBM growth. Recent data suggest that BMDCs recruitment was enhanced following therapies, e.g., anti-tumor chemotherapy or antiangiogenic therapy. The tumor-promoting myeloid cells are the subpopulations of recruited BMDCs in the microenvironment. Here, we propose the combination of anti-CSF1R, anti-CYP4A, or immune therapy with standard therapies to overcome the myeloid cell-mediated resistance in GBM.

Initially, AAT targeting the VEGF–VEGFR pathway was proposed against GBM tumors due to their hypervascular nature. However, AAT resulted in leaky blood vessels, which enhanced hypoxia and activated alternate mechanisms through recruiting BMDCs and myeloid precursors to the tumor. Some reports including publications from our laboratory identified that AAT enhanced GBM growth after transient benefits [39,50]. These results indicate that targeting the TME through myeloid inhibitor and other immune therapies is a better option that could block alternative mechanisms and will provide a benefit in anti-tumor responses. Some of these potential therapies are under investigations at the preclinical level (e.g., anti-cytochrome P450 (CYP) 4A or HET0016), early phases of clinical trials (e.g., anti-CSF1R), and others are advancing to late phase (e.g., immune therapies). There are upcoming therapies against GSCs and pericytes, key mediators of TME. However, this article only focuses on immune therapies with the special interest in targeting protumor myeloid cells in GBM tumors. Immunotherapies including anti-myeloid treatments mainly rely on polarizing the protumor microenvironment into antitumor phenotypes without disrupting the microenvironment, which is prone to hypoxia and vasculature leakiness. Moreover, this section is dedicated to potential emerging approaches and therapeutic modalities for the combination therapy options with the available standard therapies against GBM (Figure 1).

### 3.1. Anti-Myeloid Therapies

There is a significant role of TME in the modulation of therapeutic responses [106,107]. The TME in GBM tumors is characterized by active immunosuppressive mechanisms. Previously, our laboratory reported that AAT induces marked hypoxia in the invasive tumor [31], which promoted accumulation of BMDCs in GBM models [39,86]. The myeloid subpopulations of BMDCs not only suppress immune responses but also enhance neovascularization and modulate cancer stem cells (CSCs) [108,109,110,111,112,113,114]. One class of the critical tumor-associated myeloid cells is the immunosuppressive myeloid-derived suppressor cells (MDSCs), which are abundant in the GBM. MDSCs inhibit T-cell-mediated anti-tumor immunity and contribute to therapy resistance [86,115,116,117]. There are several studies showing the role of tumor-associated myeloid cells (TAMCs) in resistance to a different class of adjuvant therapies [86]. Studies noticed that tumor refractoriness to AATs was mediated by immune suppressive myeloid cells [118,119,120]. The mechanistic study identified that T helper type 17 cells and MDSCs induce the expression of granulocyte colony-stimulating factor CSF (G-CSF) in the stromal compartment, which in turn attracts MDSCs to drive anti-VEGFA resistance [121]. Similarly, we also found that myeloid cells mediate escape from AAT in a preclinical chimeric mouse model of GBM [39]. Key chemokines, such as macrophage colony stimulating factor-1 (M-CSF/CSF1) and monocyte chemotactic protein-1 (MCP1/CCL2), are known to contribute to the recruitment of myeloid cells to the tumors due to the presence of CSF1R [116,122,123]. The CSF1R expression has been reported on MDSCs, tumor-associated macrophages (TAMs), and dendritic cells [124,125,126] and regulates survival, differentiation, and proliferation of monocytes and macrophages [127,128] and has a critical role in angiogenesis and tumor progression [129,130]. Studies indicated that TME-accumulated TAMs can be targeted through anti-CSF1R with short-term treatment protocol to inhibit GBM progression via inhibiting chemokines such as CXCL7 (human IL8 variant) in animal models [39,86,131]. The CSF1R blockade has been shown to reverse macrophage polarization, inhibited GBM progression [131], and improved efficacy of RT [125]. A recent study identified that although overall survival was significantly prolonged in response to long-term CSF-1R inhibition, a subset of tumors recurred in a GBM model [132]. Consequently, combining IGF-1R or PI3K blockade with continuous CSF-1R inhibition in recurrent tumors significantly prolonged overall survival [132]. Several anti-myeloid therapies are undergoing clinical trials [133]. Therefore, targeting of tumor-recruited myeloid cells is a creative choice to target GBMs.

### 3.2. Immune Therapies

Immune therapies are a powerful strategy that inhibits tumor growth through enhancing immune responses inhibit tumor growth [134,135]. Immune therapy has two components referred to as active and passive approaches. The active immune therapy approach uses peptide or cellular vaccine to enhance the Th1 responses. However, the passive immune therapy uses the adoptive transfer of effector immune cells to induce antitumor responses [135]. Recently, several immune therapies against malignant cancers have been approved by the US FDA against malignant cancers [136]. Surprisingly, this class of therapy also showed resistance characterized by lack of durable and sustained immune responses in tumors [137,138,139] and indicated the use of the combinatorial approach, discovery of next-generation immune therapies, and anti-inflammatory approaches. Since GBM harbors a significant immune component, several immune therapy clinical trials are underway such as monotherapy or combination therapies of immune checkpoint inhibitors, peptide and dendritic cell vaccines, and adoptive T cell therapy in new or recurrent GBM tumors. Recently, Huang et al. reviewed current advances and discussed ongoing potential trials involving GBM patients [140].

### 3.3. Anti-CYP4A Therapy

Several alternative therapies are possible to overcome AAT resistance. Our research group has been studying the role of *N*-hydroxy-*N*′-(4-butyl-2 methyl phenyl) formamidine (HET0016) as a selective inhibitor of 20-HETE synthesis. 20-HETE, an arachidonic acid metabolite, can be synthesized by enzymes from the CYP4A and CYP4F families and promotes tumor neovascularization [49,141], proliferation [142], migration [143,144] and regulation of EPCs [145]. Previously, we demonstrated that the overexpression of 20-HETE can increase the GBM tumor volume by 10 fold [141]. Moreover, we observed that HET0016 controls tumor growth and migration in a time-dependent manner and attenuates the resistance of AATs [143]. Recently, we observed that VM can drive AAT resistance in GBM and the HET0016 treatment reduced the incidence of VM in a human GBM animal model [49] by altering the tumor vascular kinetics and permeability [146]. HET0016 decreased the periodic acid Schiff (PAS)-positive VM structures both at the core and the periphery of tumors thereby opening new avenues to counter AAT resistance in GBM [49].

Several other studies have shown that resistance to AAT also has a strong contribution of immune cells [81,147,148,149]. In particular, myeloid cells recruited to the TME can acquire endothelial signatures (CD202b and CD34) after vatalanib treatment [39], increasing the tumoral angiogenic potential and the resistance to treatment. In glioma, AATs were associated with the increased myeloid cell infiltration and stem cell accumulation [120]. Our laboratory has recently shown that HET0016 decreased the granulocytic MDSC (gMDSC) population in the metastatic niche by inhibiting the polarization of the MDSCs to a granulocytic phenotype [144]. A novel flavonoid, FLA-16, normalized the tumor vasculature through inhibiting CYP4A pathways and improved survival. It was accompanied by the decreased secretion of 20-HETE, VEGF and transforming growth factor (TGF)-β molecules in stroma [150]. Altogether, CYP4A expression in TAMCs is crucial for tumor dependent macrophage phenotype shift, and its inhibition by HET0016 or FLA-16 decreased tumor-associated phenotypes in GBM [150,151]. Numerous mechanisms might be involved in the 20-HETE inhibition. We previously demonstrated that HET0016 decreased proliferation of 9L gliosarcoma cells by 55% by reducing the phosphorylation of protein kinases and growth factors such as ERK1/2, stress-activated protein kinase (SAPK), cJUN, epidermal growth factor (EGF) and platelet-derived growth factor receptor (PDGFR) [142]. Moreover, we observed that HET0016 decreases expression of VEGF via ERK1/2 [141], and MMP2 and MMP9 via PI3K/AKT pathways [144]. A schematic of the signaling pathway presented by Shankar et al. [143] from our research group summarizes the possible therapeutic actions of HET0016. Given the involvement of HET0016 in decreasing the gMDSC population and phenotype shift in macrophages from M2 to M1, it is interesting to consider the role of the CYP4A/20-HETE axis in the recruitment, proliferation, and polarization of the aforementioned pro-tumorigenic immune cells. Moreover, our observations strongly support the critical role of the CYP4A/20-HETE axis in mediating AAT resistance by favoring the acquisition of endothelial signatures by myeloid cells in the TME. This evidence encourages us to exploit the CYP4A/20-HETE axis as a potential target for future therapeutic interventions [152].

## 4. Conclusions and Future Directions 

Therapy resistance is an emerging hallmark and daunting outcome for any adjuvant treatment in the clinic, and involves (1) targeting tumor cells through chemotherapies [86,153], (2) targeting endothelial cells through AATs [5,86,154], and (3) improving anti-tumor immunity through immune-therapies [138,139]. Most of the adjuvant treatments have witnessed resistance through the involvement of BMDCs and tumor-promoting myeloid cells [86,116]. Involvement of BMDCs in cancer therapy warrants a need for a careful therapeutic strategy that will not activate alternative signaling pathways of vasculogenesis via increased infiltration of myeloid cells to the tumor or tumor cell-derived VM in GBM [50,51,155]. Therapies targeted against the TME represent a promising approach for GBM therapy. Targeting the TME may have decreased likelihood of acquired resistance through mutations in the cellular microenvironment, as is frequently observed with tumor cell-targeted therapies. Since several therapies targeting the TME are undergoing clinical trials such as anti-CSF1R [133,140,156], the combination of TME-targeted therapy with available standard therapies is an ingenious choice at present to target new and recurrent GBMs. Some novel treatments that have given successful results and survival benefits in preclinical models of GBM, such as anti-CYP4A or HET0016, could be the perfect adjuvant therapy following standard therapies in GBM clinical trials in the near future [146]. Above all, GBM research laboratories are rare around the world and there is a great demand for GBM-dedicated laboratories to develop better preclinical in vivo models with the intact immune system to test novel therapies before they progress to clinical trials [157,158,159]. 

## Figures and Tables

**Table 1:** A list of critical protein molecules involved in the GBM microenvironment.

Key Protein	Full Name	Category	Key Function(s)
ANGPT2	Angiopoietin-2	Growth factor	Tumor neovascularization, metastasis, and inflammation
COL1A1	Collagen, type 1, alpha (α) 1	Structural protein, part of connective tissue	Tumor neovascularization
CD31/PECAM-1	Platelet endothelial cell adhesion molecule	Endothelial cell marker	Leukocyte transmigration, neovascularization, and integrin activation
CD34	Hematopoietic progenitor cell antigen	Hematopoietic stem cell marker	Attachment of stem cells to bone marrow ECM, stromal cells, facilitates cell migration
CD45	Protein tyrosine phosphatase, receptor type, C (also known as Common leukocyte antigen)	Pan-leukocyte marker	Signal transduction in hematopoiesis
CD133	Prominin-1	Stem-cell marker	Cancer stem cells with CD133 undergo self-renewal and differentiation
CD202b	Angiopoietin-1 receptor	Endothelial-cell marker	Promotes neovascularization
CSF	Colony-stimulating factor 1/Macrophage colony-stimulating factor	Cytokine	proliferation, differentiation, and survival of monocytes, macrophages, and bone marrow progenitor cells
CSF-1R	Colony-stimulating factor receptor-1	Cytokine receptor	Cytokine receptor that facilitates the actions of CSF-1
CYP4A and CYP4F	Cytochromes P450 family of enzymes	Enzymes involved in arachidonic acid metabolism	Production of 20-HETE, an eicosanoid metabolite that promotes neovascularization, migration, inflammation, and metastasis
CXCL7	Chemokine (C-X-C motif) ligand 7	Cytokine	mitogenesis, synthesis of extracellular matrix, glucose metabolism and synthesis of plasminogen activator, recruitment of CXCR2+ myeloid cells
CXCL8 (IL-8)	Chemokine (C-X-C motif) ligand 8 (Interleukin-8)	Chemokine	Neutrophil chemotactic factor, chemotaxis of other granulocytic cells and CXCR2+ myeloid cells, potent pro-neovasculogenic chemokine
EGF	Epithelial Growth Fator	Growth factor	Cellular proliferation, differentiation, and survival
Eph A1 and A2	Ephrin A1 and A2	Receptor tyrosine kinase	Embryonic development, post-natal angiogenesis, stem cell differentiation and migration
FGF	Fibroblast growth factor	Growth factor	Angiogenesis, wound healing, embryonic development, and various endocrine signaling pathways, proliferation, and differentiation of various cell types
G-CSF	Granulocyte-colony stimulating factor	Cytokine	Survival, proliferation, differentiation, and function of neutrophil precursors and mature neutrophils
HGF	Hepatocyte growth factor	Growth, motility and morphogenic factor	Embryonic organ development, specifically in myogenesis, in adult organ regeneration, and in wound healing, mediates pro-tumorigenic roles in growing tumors
HIF-1α	Hypoxia-inducible factor 1 α	Transcription factor	Released in response to hypoxia, neovascularization, energy metabolism, cell survival, and tumor invasion
IGF	Insulin-like growth factor	Growth factor	Promotes growth and survival of tumor cells
MCP-1/CCL2	Monocyte chemoattractant protein 1	Chemokine	Recruitment of several inflammatory monocytes, memory T cells, and dendritic cells to the tumor
MGMT	*O*-6-methylguanine-DNA methyltransferase	Enzyme/protein	DNA Repair promotes resistance of tumor cells to chemotherapy (esp. Temozolomide (TMZ))
MMP-2 and 9	Matrix Metalloproteinases-2 and 9	Proteinase enzymes	Degradation of extra-cellular matrix (ECM) proteins, promotes angiogenesis by ECM remodeling
NG2	Neuron-glial antigen 2/Chondroitin sulfate proteoglycan 4	Chondroitin sulfate proteoglycan	Tumor cell metastasis and invasion
PDGF	Platelet-derived growth factor	Growth factor	Pro-angiogenic molecule
PLGF	Placental growth factor	Growth factor	Pro-angiogenic molecule
SDF-1α	Stromal-derived factor 1 α	Chemokine	Chemotactic protein to facilitate recruitment of bone marrow-derived cells and endothelial progenitor cells
VEGF	Vascular endothelial growth factor	Growth factor	Promotes neovascularisation by facilitating survival and development of endothelial cells and proliferation of endothelial progenitor cells
VEGFR	Vascular endothelial growth factor receptor	Receptor tyrosine kinase	Receptor for VEGF to promote neovascularization
TN-C	Tenascin C	Glycoprotein	Tumor cell proliferation and migration
20-HETE	20-Hydroxyeicosatetraenoic acid	Eicosanoid metabolite of Arachidonic acid	Neovascularization, tumor cell growth, proliferation, migration, and recruitment of angiogenic myeloid cells to tumor microenvironment

## Abbreviations

20-HETE	20-Hydroxyeicosatetraenoic acid
CD202b	Angiopoietin-1 receptor
AGPT2	Angiopoietin-2
AAT	Antiangiogenic therapy
BMDCs	Bone marrow-derived cells
CSC	Cancer stem cell
CBTRUS	Central Brain Tumor Registry of the United States
CNS	Central nervous system
CXCL7	Chemokine (C-X-C motif) ligand 7
CXCL8	Chemokine (C-X-C motif) ligand 7
CD	Clusters of differentiation
COL1A1	Collagen, type 1, alpha 1
CSF	Colony-stimulating factor
CSF1R	Colony-stimulating factor receptor 1
CD45	Common leukocyte antigen
CXCR4	C-X-C Motif Chemokine Receptor 4
CYP4A and CYP4F	Cytochromes P450 family enzymes
DLL	Delta-like ligand
ECs	Endothelial cells
EPCs	Endothelial progenitor cells
Eph	Ephrin
EGF	Epidermal growth factor
EGFR	Epidermal growth factor receptor
FGF	Fibroblast growth factor
GBM	Glioblastoma
GBM-O	Glioblastoma with oligodendroglioma component
GSCs	Glioma stem cell-like cells
G-CIMP	Glioma-CpG island methylator phenotype
G-CSF	Granulocyte-colony stimulating factor
gMDSC	Granulocytic myeloid-derived suppressor cells
GFP	Green fluorescent protein
CD34	Hematopoietic progenitor cell antigen
HGF	Hepatocyte growth factor
hTERT	human telomerase reverse transcriptase
HIF1-α	Hypoxia-inducible factor- alpha
IGF	Insulin-like growth factor
IL8	Interleukin 8
IDH	Isocitrate dehydrogenase
MMPs	Matrix metalloproteinases
MAPK	Mitogen-activated protein kinase
MCP-1	Monocyte chemoattractant protein 1
MDM2	Mouse double minute 2 homolog
MDSC	Myeloid-derived suppressor cells
NG2	Neuron-glial antigen 2
HET0016	*N*-Hydroxy-*N*′-(4-butyl-2-methylphenyl)-formamidine
NO	Nitric oxide
NFκB	Nuclear factor-kappa beta
MGMT	*O*-6-Methylguanine-DNA Methyltransferase
PAS	Periodic Acid Schiff
PTEN	Phosphatase and tensin homolog
PI3K	Phosphatidylinositide 3-kinase
PLGF	Placental growth factor
CD31/PECAM-1	Platelet endothelial cell adhesion molecule
PDGF	Platelet-derived growth factor
PDGFR	Platelet-derived growth factor receptor
RT	Radiotherapy
CD133	Stem-cell marker (Prominin-1)
SDF-1α	Stromal-derived factor 1 alpha
TMZ	Temozolomide
TN-C	Tenascin C
TGFβ	Transforming growth factor-beta
TME	Tumor microenvironment
TP53	Tumor p53
TAM	Tumor-associated macrophage
TAMCs	Tumor-associated myeloid cells
TDEC	Tumor-derived endothelial cells
US FDA	United States Food and Drug Administration
VEGF	Vascular endothelial growth factor
VEGFR	Vascular endothelial growth factor receptor
VM	Vascular mimicry
WHO	World Health Organization
